# Extrinsic Calibration of 2D Laser Rangefinders Using an Existing Cuboid-Shaped Corridor as the Reference

**DOI:** 10.3390/s18124371

**Published:** 2018-12-10

**Authors:** Deyu Yin, Jingbin Liu, Teng Wu, Keke Liu, Juha Hyyppä, Ruizhi Chen

**Affiliations:** 1 State Key Laboratory of Information Engineering in Surveying, Mapping and Remote Sensing, Wuhan University, Wuhan 430079, China; deyu.yin@whu.edu.cn (D.Y.); whurswuteng@whu.edu.cn (T.W.); kekeliu@whu.edu.cn (K.L.); ruizhi.chen@whu.edu.cn (R.C.); 2 Collaborative Innovation Center of Geospatial Technology, Wuhan University, Wuhan 430079, China; 3 Department of Remote Sensing and Photogrammetry, Center of Excellence in Laser Scanning Research, Finnish Geospatial Research Institute, 02430 Masala, Finland; juha.hyyppa@nls.fi

**Keywords:** 2D laser rangefinder, extrinsic calibration, mobile mapping, indoor positioning, line detection

## Abstract

Laser rangefinders (LRFs) are widely used in autonomous systems for indoor positioning and mobile mapping through the simultaneous localization and mapping (SLAM) approach. The extrinsic parameters of multiple LRFs need to be determined, and they are one of the key factors impacting system performance. This study presents an extrinsic calibration method of multiple LRFs that requires neither extra calibration sensors nor special artificial reference landmarks. Instead, it uses a naturally existing cuboid-shaped corridor as the calibration reference, and it hence needs no additional cost. The present method takes advantage of two types of geometric constraints for the calibration, which can be found in a common cuboid-shaped corridor. First, the corresponding point cloud is scanned by the set of LRFs. Second, the lines that are scanned on the corridor surfaces are extracted from the point cloud. Then, the lines within the same surface and the lines within two adjacent surfaces satisfy the coplanarity constraint and the orthogonality constraint, respectively. As such, the calibration problem is converted into a nonlinear optimization problem with the constraints. Simulation experiments and experiments based on real data verified the feasibility and stability of the proposed method.

## 1. Introduction

A 2D laser rangefinder (LRF) can provide an accurate range with high angular resolution over long distances [1]. As it has lower power consumption, smaller size, and lower cost compared to general 3D laser scanners, it is widely used in autonomous systems such as robot positioning [2], navigation [3], and mobile mapping [4,5] through the simultaneous localization and mapping (SLAM) technique [6]. Many systems, such as the light detection and ranging (LiDAR)-based SLAM system [7,8], employ several 2D LRFs simultaneously to perform sensor fusion, which reinforces the concern of extrinsic calibration for fusing all the LiDAR point cloud into a global reference frame. Moreover, the performances of mapping and SLAM are sensitive to calibration errors, especially when they have a long working range, so that small rotation errors can produce significant distortions in the map [9].

The calibration of the sensors is generally divided into intrinsic parameter calibration and extrinsic parameter calibration. The intrinsic parameters relate to the acquisition process and involve issues that are both temporal and geometric [10,11]. The extrinsic parameters determine the pose transformation relationship among multiple sensors or between one sensor and a reference coordinate system [12]. The work of this paper focuses on the extrinsic calibration of multiple 2D LRFs, assuming that the intrinsic calibration has been done.

The extrinsic calibration of multiple 2D LRFs is more difficult than the extrinsic calibration of multiple 3D laser scanners [13,14], multiple depth cameras [15], or even the extrinsic calibration between one LRF and one camera [16,17,18]. The latter can be done by placing retroreflective targets or using features that are easily recognized in 3D scenes, whereas it is hard to find discriminative features in a 2D LRF point cloud. Although multiple LRFs can be indirectly calibrated by doing the calibration between each LRF and another type of sensor, such as a camera, not all LRFs have enough of a common field of view with the camera, and the propagation of error can also affect the final calibration accuracy.

Some applications may only be concerned with three degrees of freedom (DOF) of external reference calibration on the horizontal scanning plane. For example, in References [19,20], the three-DOF extrinsic calibration of multiple LRFs on the scanning plane was achieved by matching the target motion trajectories in the overlapping scanning region of the LRFs. However, for many applications, such as 3D mapping applications, there may be large angles between scanning planes [8,12], and then a six-DOF extrinsic calibration needs to be done. The authors of Reference [12] made a special facility based on the scanning planes of multiple LRFs to ensure all the LRFs were able to scan the small V-shaped targets mounted on the facility, and then they used the centers of the targets as control points for the extrinsic calibration. Obviously, this method required manual targets to be made based on pre-measured installation configurations, and this method did not automatically solve the problem of finding distinguishing features on multiple LRF scan planes.

However, although it is difficult to directly find corresponding discriminative features in LRF point clouds, some geometric characters can be used to indirectly estimate the control points located outside the scanning planes of LRFs. As well as using spherical targets to do an extrinsic calibration of multiple Kinect sensors [21], the 2D LRF can obtain a circular arc when scanning a spherical target, the position of the spherical center outside of the scanning plane can be derived according to the known radius of the target, and then the extrinsic calibration between multiple LRFs can be performed using the corresponding target centers in all the frame data [22,23]. In Reference [24], the extrinsic calibration between a single LRF and a fixed 3D reference frame was done by designed targets using the inherent geometrical characteristics formed by a cone and a pyramid. Additionally, the authors in Reference [25] used conic targets to perform an extrinsic calibration between a 3D laser scanner and a 2D LRF. In References [26,27,28], geometric-based methods were used in the extrinsic calibration between a single LRF and a camera. Reference [26] used a scene corner to form a line-to-plane and a point-to-plane constraint, Reference [27] used a simple folding pattern to form a rotation constraint and a point-to-plane constraint, and Reference [28] used a V-shaped pattern to form a point-to-plane constraint. All these geometric constraints can convert the calibration problem to an error minimization problem so as to achieve extrinsic calibration.

In Reference [29], the authors used the geometric constraints formed by static objects (buildings) to achieve the extrinsic calibration between a 3D laser scanner and an inertial measurement unit (IMU) sensor. In References [30,31,32], the calibration between a 2D LRF and an IMU, and a GNSS and a body reference frame of the multisensor system, was deployed by using differently orientated georeferenced planar surfaces. With the linking component from a 3D scanner, the calibration could be done by minimizing distance between the pointwise-observed referenced surfaces and their nominal position. If this method were used for calibrating the 2D LRFs, however, multiple line segments could be gotten instead of line-plane pairs, so the problem would be totally different.

According to the literature surveyed, in recent years some research works have directly calibrated the extrinsic parameters of multiple LRFs based on geometric constraints, and because the geometric constraints they used could be found in daily life, the man-made scanning target was omitted. References [33,34] used a coplanarity constraint and an orthogonality constraint formed by a pair of perpendicular planes for the extrinsic calibration of LRFs. Generally, this geometric pattern can be found in daily life, such as the corner portion of a vertical wall outside a building. The author of Reference [35] used two kinds of coplanarity constraints formed by a large plane to do the extrinsic calibration of LRFs. This method requires a flat surface that is large enough and flat enough, such as a playground floor. It requires at least three LRFs. According to our practical experience, for the methods in References [33,34], it is difficult to find a corner that is large enough, free of obstacles, and not made of reflective or light-transmissive glass in our modern living environment. This paper proposes a method using the same geometric constraints but choosing a common cuboid-shaped corridor as the georeferenced target. That is, the method does not need extra sensors or artificial landmarks, and it utilizes a coplanarity constraint and an orthogonality constraint formed by a corridor, rotating the multiple LRFs with different orientations to perform the extrinsic calibration.

The rest of the paper is organized as follows: Section 2 introduces the basic calibration principle of this method; Section 3 describes the detail of the calibration methodology; Section 4 shows the simulation experiments and the experiments based on real data; and Section 5 and Section 6 are the discussion and the conclusion of this work.

## 2. Calibration Principle

This section explains the objective of an extrinsic calibration, the reason why a cuboid-shaped corridor can be used to do the calibration, and the calibration procedures.

### 2.1. Objective

**Figure 1:**
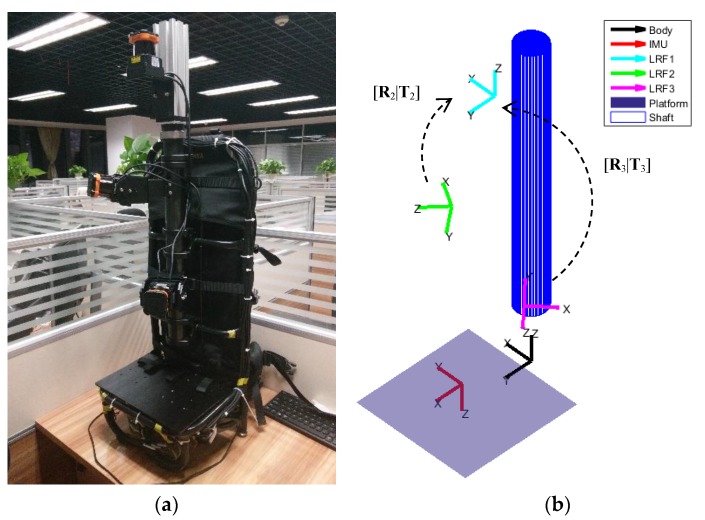
The indoor mapping device: (**a**) Picture; (**b**) frames in the device.

An indoor mapping device was taken as an example, as shown in Figure 1. It is a laser-based backpack and trolley device. Three 2D LRFs and one inertial measurement unit (IMU) were installed on the device, and their own coordinate frames are shown in Figure 1b. For the device, the extrinsic parameters of the four sensors needed to be calibrated, but only the calibration of the three LRFs is discussed here: It was also planned to be the premise of the IMU extrinsic calibration. Thus, the objective of the extrinsic calibration was to obtain the relative rotation and translation parameters between the three LRFs.

The three LRFs are denoted as LRF_1_, LRF_2_, and LRF_3_ from top to down, and the coordinate frames of them are denoted as S_1_, S_2_, and S_3_. Let [**R**_1_|**T**_1_], [**R**_2_|**T**_2_], [**R**_3_|**T**_3_]$\in {ℜ}^{3}$ be the LRF poses with respect to a common reference frame. For the hardware system, the frame of the IMU may be used as a device frame and also as a reference frame, but for the convenience of calibration, S_1_ is taken as the reference frame. So, the final calibration results are [**R**_2_|**T**_2_] and [**R**_3_|**T**_3_], and in terms of values are three Euler angles and three translation values for each of [**R**_2_|**T**_2_] and [**R**_3_|**T**_3_].

### 2.2. Geometric Constraints

**Figure 2:**
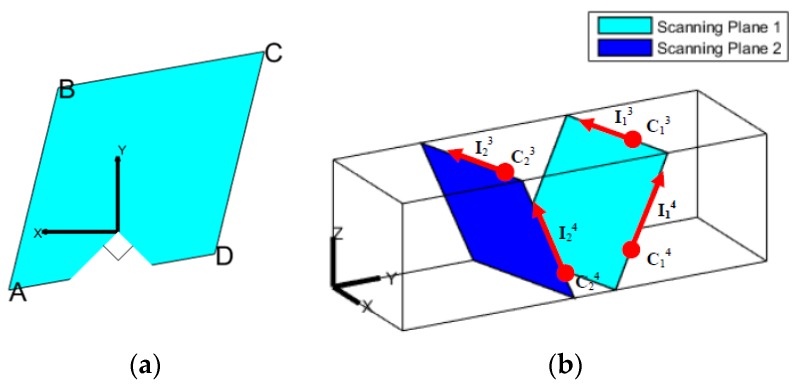
The schematic of scanning the cuboid-shaped corridor with laser rangefinders (LRFs): (**a**) The 2D view of the scanned parallelogram by a single LRF; (**b**) the 3D view of the two scanned parallelograms scanned by two LRFs.

The scanning plane of each LRF is a 2D plane in the 3D real word. Ideally, when it is used to scan a cuboid-shaped corridor, a parallelogram is gotten, as shown in Figure 2a (the 90° dead zone of the LRF should be noted, and the *z* axis is behind the 2D figure, actually, according to the right-hand rule).

When scanning the cuboid-shaped corridor using multi-LRFs, multiple parallelograms can be gotten, and all the sides of the parallelograms are on the surfaces of the corridor. As shown in Figure 2b, $C_{i}^{a}$ and $I_{i}^{a}$ are the center points and the line vectors of the lines scanned by LRF*_i_* (*i* = 1, 2, 3; *a* = 1, 2, 3, 4) on the surface *a*. After converting these scanned 2D lines into 3D space, there are two types of geometric constraints, the coplanarity constraint and the orthogonality constraint.

The coplanarity constraint means that the lines scanned by LRFs, which lie on the same surface, should be on the same 3D plane. That is, the distance between the two coplanar lines should be zero. Thus, the coplanarity constraint is expressed as follows:

(1) $$(R_{1}I_{1}^{a}\times R_{2}I_{2}^{a})\cdot (R_{1}C_{1}^{a}+T_{1}-R_{2}C_{2}^{a}-T_{2})=0,$$

where a denotes the surface the lines are on. The first-half part of the left item of the equation can represent the distance vector between them, and then the left item of the equation equals the distance value. 

The orthogonality constraint means that two adjacent corridor surfaces should be perpendicular to each other, whereas the normal vector of the surface can be expressed by the cross product of the line vectors. Thus, the orthogonality constraint is expressed as follows:

(2) $$n^{a}\cdot n^{b}=(R_{1}I_{1}^{a}\times R_{2}I_{2}^{a})\cdot (R_{1}I_{1}^{b}\times R_{2}I_{2}^{b})=0,$$

where a and b denote two adjacent surfaces of the corridor. Generally, the cross product of vectors of two lines can be taken as the normal vector of the plane they are on. Then the perpendicular relationship of the two planes means that the dot product of their normal vectors should be zero.

The geometric constraints are the key point of the calibration, and the initial poses of LRFs are given at first. Because they deviate from the correct poses, the scanned lines do not satisfy the coplanarity constraint and orthogonality constraint. The calibrated poses can be gotten by finally minimizing this deviation.

However, obviously, the extrinsic calibration of a LRF is a nonlinear problem with six DOFs, whereas just one corridor observation cannot provide full six DOF constraints. As shown in Figure 2b, assume the calibration is done with all the lines meeting the geometric constraints using just one observation, and the translation parameter along the *y* axis is still uncertain. Therefore, in order to provide sufficient constraints by the corridor observations, the device must be required to gather data while rotating inside the corridor with different orientations. The specific rotating operation will be stated and discussed in Section 4.1 and Section 5.

### 2.3. Calibration Procedure

The entire calibration solution consists of three key points: (1) Line detection; (2) the acquiring of coplanar line pairs and orthogonality line pairs with respect to the coplanarity constraint and the orthogonality constraint, respectively; (3) solving the calibration problem with these line pairs as input.

**Figure 3:**
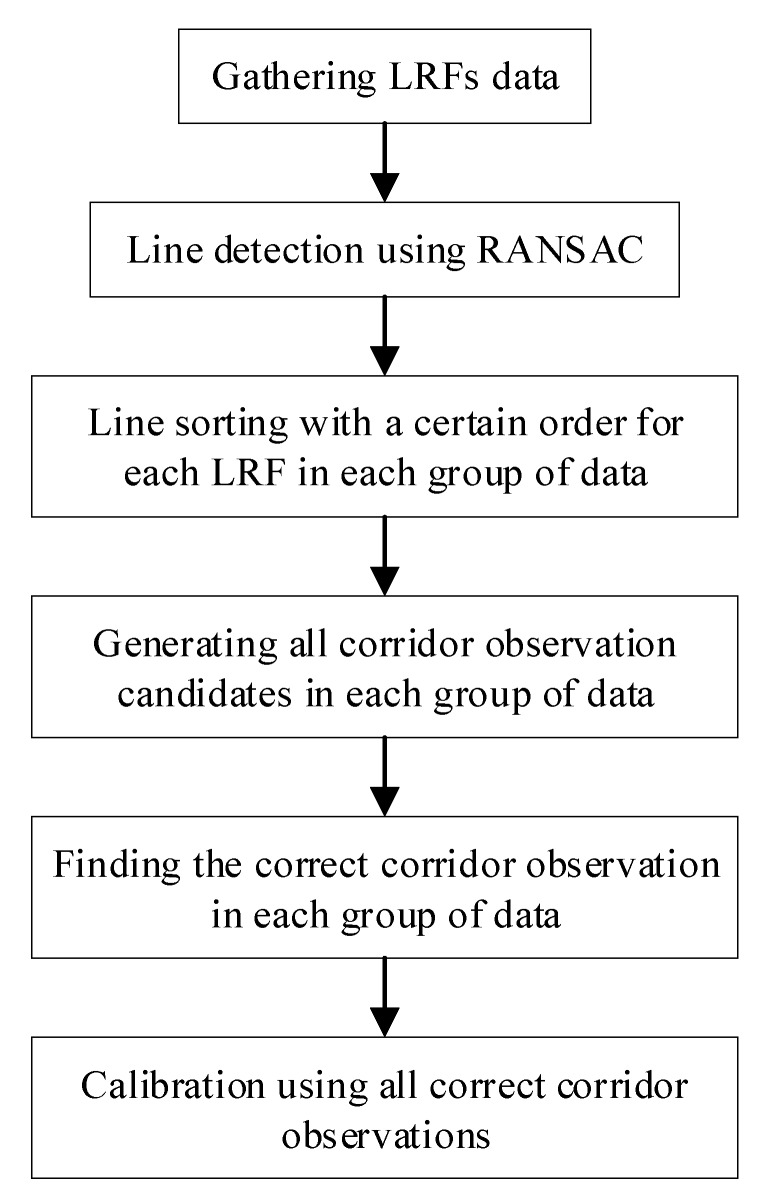
Calibration procedure.

After the scanned lines are detected from the raw LRF data, it is still unknown which lines lie on the same corridor surface as well as which coplanar line pairs lie on two neighboring corridor surfaces, even when the initial poses of LRFs are given at first. In fact, the problem of getting these line pairs means finding the correct corridor observation based on each group of data. Hence, the line sorting method, the generating of all corridor observation candidates, and the method of finding correct corridor observations were designed to solve this problem. The whole calibration procedure is shown in Figure 3. The detailed explanation of these processes is carried out in Section 3.

## 3. Method

### 3.1. Line Detection

A random sample consensus (RANSAC) [36] is used to detect the lines in the 2D points scanned by LRFs. As can be seen in Figure 2a, there exists a discontinued line (line “A–D”) due to the dead view zone of LRFs (or due to other reasons such as reflection), and this can be clustered as the same line by RANSAC.

In order to enhance the robustness of detecting lines using RANSAC, some modifications were made on the basis of traditional algorithms:

(1) The nearest available distance threshold was added. If a person is holding a device to rotate a device equipped with LRFs during the experiment, it is possible to scan a portion of the human body that is closer to the distance, and the nearest available distance threshold according to the calibration scene can filter out such interference.

(2) The farthest available distance threshold was added. First, the common LRF sensors can scan to a distance of tens of meters. If the calibrated scene has only a limited distance or the further scene is too messy, then it is necessary to add the farthest available distance threshold to avoid impact from the further scene. Second, considering the issue of balance (related to the methods in Section 3.4), the detected lines are not allowed to be too long.

(3) The minimum limit of the distance between two sampled points was added. Assuming that the two sampled points are on the same line, the greater the distance between them, the closer the line is to the actual line. On the other hand, it is also considered that when the LRF scans a close-range target, the point density is relatively large due to the fixed angular resolution, and even a short line may have many inliers, so this limitation also limits the interference of the near object to the line detection to some extent.

(4) Traditional RANSAC saves the model whenever it finds enough inliers. However, considering multiple lines should be detected, and inliers are not shared between them, an inner loop with a fixed number of repetitions is added to ensure that lines with more inliers have a higher extracted priority.

The line detection algorithm is shown in Algorithm 1.

**Algorithm 1.** Line detection based on modified random sample consensus (RANSAC).
**Input:**

  2D points: $P=\left\{p_{i}\right\}=\left\{x_{i},y_{i}\right\}$  Nearest valid distance: NVD  Farthest valid distance: FVD  Threshold used to differentiate between inliers and outliers: ε  Shortest line length: SLL  Minimum number of inliers: MNI  Maximum outer loop times: MOLT  Fixed inner loop times: FILT
**Output:**

  The number of lines: nL  All the lines: Ls  The inliers for each line: Inliers
**Procedure:**

  (1) SC← Remove the points that are too near (using NVD) or too far (using FVD) from P;  (2) Repeat sampling two points $p_{1}$ and $p_{2}$ from SC within FILT times until the distance between $p_{1}$ and $p_{2}$ is bigger than SLL;  (3) Generate a 2D line model based on $p_{1}$ and $p_{2}$: $\;\left\{A,B,C\right\}\;\left(Ax+By+C=0,\;A^{2}+B^{2}=1\right)$;  (4) Get the number of points to satisfy |Ax+By+C|<ε (inliers) in SC;  (5) Repeat (2), (3), and (4) for FILT times, and get the line L with the biggest number of inliers;  (6) If the number of inliers based on L is bigger than MNI, then $L^{*}\leftarrow$ refine L with the least-square method and recompute the inliers based on $L^{*}$, else return all the lines and the number of them now;  (7) Save $L^{*}$ to Ls and save the inliers to Inliers;  (8) SC← Remove the inliers from SC based on model $L^{*}$;  (9) Repeat (2)–(8) until the number of points in SC is less than MNI or the number of repetitions is greater than MOLT;  (10) Return the number of lines nL, all the lines Ls, and Inliers.

### 3.2. Line Sorting

The lines detected by the method based on modified RANSAC are unordered with respect to the real physical corridor surfaces. The purpose of line sorting is to sort these lines with respect to the thee-surfaces order and to reduce the complexity of finding the correct corridor observation (details are in Section 3.3 and Section 3.4). This means each line is set a line index. Suppose there are n (n≤4) straight lines, then one of them is chosen to be the first line, and other lines are set an index between 2 and 4 with respect to the physical order.

**Figure 4:**
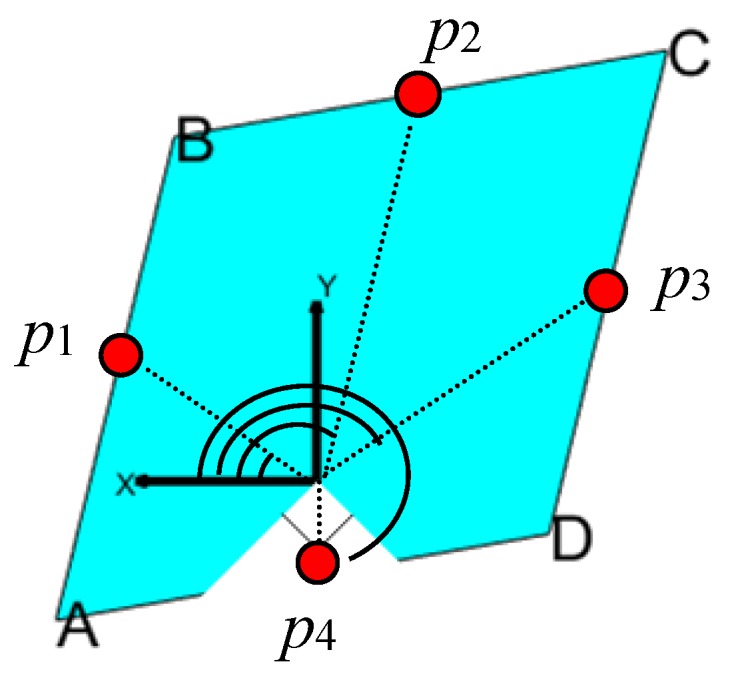
The schematic of line sorting. The solid red circles denote the center points of the lines.

The line sorting method is applied as follows:

(1) compute the center points of each line;

(2) sort the lines by the angle between the vector from zero point to the center point and the vector of the positive *x* axis direction (as shown in Figure 4); 

(3) set the line index of the first line as “1st”, and infer whether the line with the next index is an opposite line or the previous adjacent line or next adjacent line, and repeat this until each line is set an index.

In the third step, the relationship between two lines is inferred by the vector of the line and the vector from zero point to its center point for each line. Assume $v_{1}$ and $v_{2}$ are the two vectors of line $l_{1}$ and line $l_{2}$, whose line indexes are $i_{1}$ and $i_{2}$. If $\left|v_{1}\times v_{2}\right|\approx 0$, then the two lines are on two opposite surfaces, which means $\left|i_{1}-i_{2}\right|=2$, else they are on two adjacent surfaces. Then, if $l_{1}$ and $l_{2}$ are on two adjacent surfaces, assume $u_{1}$ and $u_{2}$ are the two vectors from zero point to the center points of $l_{1}$ and $l_{2}$. If $\left(u_{1}\times u_{2}\right)\cdot Z>0$ (Z is the vector of the positive direction of the *z* axis), then $i_{2}=mod\left(i_{1},4\right)+1$, otherwise, $i_{1}=mod\left(i_{2},4\right)+1$.

### 3.3. Generating All Corridor Observation Candidates in Each Group of Data

After the line sorting process, a sorted line set is gotten in each group of data:

(3) $$SL=\left\{SSL_{i}\right\}\;\left(i=1,2,\ldots n_{LRFs}\right),$$

where $n_{LRFs}$ is the number of LRFs, and $SSL_{i}$ is the subsorted line set, which comes from the sorted lines of LRF*_i_*,

(4) $$SSL_{i}=\left\{L_{i}^{j}\right\}\;\left(i=1,2,\ldots n_{LRFs}\right),\;$$

where $L_{i}^{j}$ denotes the sorted line with an index of j in the lines from LRF*_i_*. Each LRF can get up to 4 lines in each group of data, which is based on the fact that each LRF can get at most 1 line on each surface of the corridor. Thus, the number of elements in $SSL_{i}$ is between 0 and 4. It should be noted that the index of the lines may be not continuous. For example, if there are only two lines detected from LRF*_2_*, and they lie on opposite corridor surfaces, then $SSL_{2}=\left\{L_{2}^{1},L_{2}^{3}\right\}$, according to Section 3.2.

It is still unknown which line pairs are on the same surface as well as which coplanar line pairs lie on two neighboring corridor surfaces after the above processing. The corridor observation is used to describe this problem.

A corridor observation can be taken as a line container. Specifically, because the corridor has four surfaces, one corridor observation consists of four line sets,

(5) $$CO=\left\{S_{1},S_{2},S_{3},S_{4}\right\},$$

where $S_{a}\;\left(a=1,2,3,4\right)$ denotes the line set with respect to corridor surface a:

(6) $$S_{a}=\left\{L_{i}^{a}\right\}\;\left(a=1,2,3,4;i=1,2,\ldots n_{LRFs}\right).$$

In order to get CO, all the lines in SL need to be arranged in the four line containers of the CO. By comparing Equation (4) with Equation (6), it is found that the essence of the problem is how to convert $L_{i}^{j}$ into $L_{i}^{a}$. In other words, it is how to convert the line index into the corridor surface index with the interval of indexes staying the same. In this way, the lines with the same surface index are coplanar line pairs, and the lines with adjacent surface indexes are on two surfaces that are perpendicular to each other.

**Figure 5:**
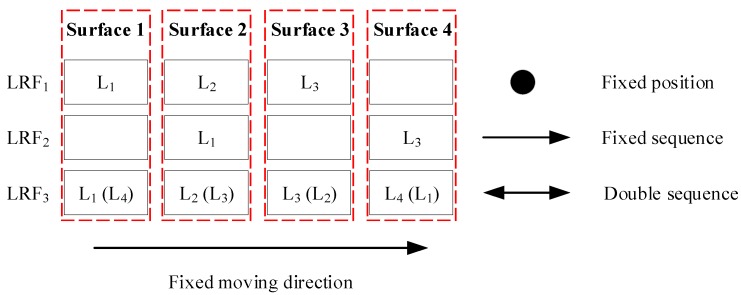
The schematic of the generating of all corridor observation candidates.

Suppose that there are 3 lines from LRF_1_, 2 lines from LRF_2_, and 4 lines from LRF_3_. If the line sequence in each LRF is not being considered, then the number of all the corridor observation candidates is $A_{4}^{3}\times A_{4}^{2}\times A_{4}^{4}$ =6912 (it is an arrangement problem, where each LRF can get at most 1 line in each surface of the corridor). However, when the line sequence is considered, and assuming the two lines in LRF_2_ lie on two opposite corridor surfaces, then the generating of all corridor observation candidates can be described as putting the lines into the four surfaces (line containers), which is shown in Figure 5.

To get all the corridor observation candidates, the surface indexes of the lines for each LRF should be moved together. If there are no adjacent lines in one LRF, such as LRF_2_ here, then the line sequence should be fixed. Otherwise, the line sequence should be double (such as LRF_3_, and the opposite sequence is L_4_, L_3_, L_2_, L_1_). However, regardless of the double sequences, the direction of movement must be fixed. Thus, there are (1×4)×(2×4)=32 (where “1” and “2” stand for a fixed sequence and double sequence, respectively) corridor observation candidates. If there exist adjacent lines of LRF_2_, the number doubles.

If there is a special case where no lines are detected in the point cloud of one of the LRFs, then this observation is abandoned. Otherwise, finally, the total number of all possible observations is as seen in Equation (7):

(7) $$\tau =4\times 2^{{ℵ}_{1}\times {ℵ}_{2}}\times 4\times 2^{{ℵ}_{3}},$$

where ${ℵ}_{i}\left(i=1,2,3\right)$ is related to whether the lines from LRF_2_ and LRF_3_ need to be moved in a double sequence. If there is one line or two opposite lines detected in LRF*_i_*, then ${ℵ}_{i}=0$. Otherwise, ${ℵ}_{i}=1$.

In addition, although the calibration is working with pairs of LRFs, considering all the lines from three LRFs into the generation makes the assessment of the corridor observation more stable.

### 3.4. Finding the Correct Corridor Observation in Each Group of Data

Among all the corridor observation candidates generated, a corridor observation assessment method based on a coplanarity assessment method is used to assess them, and the corridor observation with lowest error is taken as the correct corridor observation.

In Reference [33], the authors did the calibration by scanning perpendicular planes, which generated all the two possible corner observations for each pair of LRFs (each LRF gets two lines totally on the corner), and then they used RANSAC to filter out the incorrect corner observations based on the initialized relative poses of LRFs. This meant that at least half of the observations were totally incorrect from the original input observations, and the inliers were different at each calibration experiment even with the same data and the same parameters due to the corner observation selection method, which led to an unstable calibration result. In this method, with the line sorting result and the generating of all corridor observation candidates above, the correct corridor observation, which means all the coplanar line pairs and the neighboring surface pairs are all correct, can be gotten by using the corridor observation assessment method, which is also based on the coplanarity assessment method.

#### 3.4.1. Coplanarity Assessment Method

**Figure 6:**
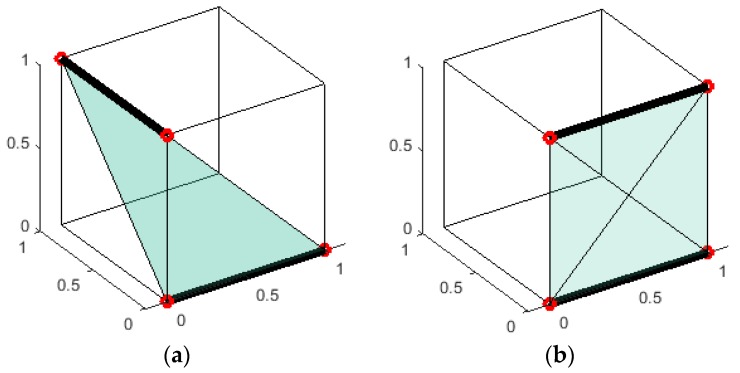
The schematic of assessing the coplanar lines by the volume of the tetrahedron. The red points are the end points of the black lines, and the transparent surfaces are the tetrahedron drawn by two end points. (**a**) A bad coplanar line pair, and the volume of the tetrahedron they form is 1/6; (**b**) a good coplanar line pair, and the volume of the tetrahedron they form is 0.

The line detection procedure generates the inliers of the lines, so it is easy to get the two end points of each line. The volume of the tetrahedron, whose vertices are the four end points of the two lines, is used to assess the coplanarity of the two lines. The two lines are more inclined to be a coplanar line pair if their tetrahedron is small, as shown in Figure 6 (the box has no relation to the method, and it is to make the figure be more like a 3D one).

Assume $\vec{a}$, $\vec{b}$, and $\vec{c}$ are the three vectors from one vertex to the other three vertices in one tetrahedron, and then the volume of the tetrahedron can be computed easily by the below equation with very little computation:

(8) $$V_{\mathrm{tetrahedron}}=\left|\frac{(\vec{a}\times \vec{b})\cdot \vec{c}}{6}\right|.$$

Considering the endpoints of the lines are the points scanned by LRFs, they may not lie on the detected lines, which can produce a small error. Thus, the endpoints in the inliers are replaced by the projection of them onto the line.

#### 3.4.2. Corridor Observation Assessment Method

With all possible corridor observations generated by the method in the previous section, each of them can be assessed by the sum of all the volume of tetrahedrons in all the four corridor surfaces:

(9) $$V_{CO}=\sum_{w=1}^{4}\Phi (l_{1},l_{2},\ldots l_{n_{w}}),$$

where $n_{w}$ denotes the number of lines in the corridor surface w, the function Φ represents the sum of the volumes of the tetrahedrons formed by all the line segment pairs that can be formed by lines from $l_{1}$ to $l_{n_{w}}$, and $V_{\mathrm{CO}}$ is the final assessment score of the corridor observation.

The coplanarity assessment score represents the error formed by the condition that the two lines are taken as a coplanar line pair, and the corridor assessment score represents the error formed by the condition that the line sets in CO are taken as a corridor observation. Thus, the corridor observation with the smallest sum volume of tetrahedrons is taken as the correct observation finally.

### 3.5. Calibration Using All Correct Corridor Observations

One correct corridor observation can be gotten after the assessment process based on one frame of data from all LRFs. The input of the calibration solution is a frame sequence with different poses, so many corridor observations with correct coplanar line pairs and correct perpendicular corridor surface pairs can be gotten. Finally, the calibration can be converted into a nonlinear optimization problem based on the coplanarity constraint and orthogonality constraint, which is expressed as:

(10) $$\underset{\left\{R,T\right\}}{\mathrm{argmin}}\sum_{i=1}^{N}\left(\sum_{a=1}^{4}\omega_{i}^{a}{\left(\left(R_{j}I_{j}^{a}\times R_{k}I_{k}^{a}\right)\cdot \left(R_{j}c_{j}^{a}+T_{j}-R_{k}c_{k}^{a}-T_{k}\right)\right)}^{2}+\;\sum_{a=1}^{3}\omega_{i}^{a,a+1}{\left(\left(R_{j}I_{j}^{a}\times R_{k}I_{k}^{a}\right)\cdot \left(R_{j}I_{j}^{a+1}\times R_{k}I_{k}^{a+1}\right)\right)}^{2}\right),$$

where N is the number of corridor observations, j is the index of the LRF to be calibrated, k is the index of the LRF to be referenced, a indicates the surface index of the corridor, and $\omega_{i}^{x}$ (the superindex x stands for a or {a, a+1}) is the weight of the corresponding residual from $CO_{i}$, which is computed through linearization from a first-order Taylor approximation of the error functions.

The resulting nonlinear least squares problem is solved iteratively using Levenberg–Marquardt:

(11) $${\left[\mu_{2}^{k}\Delta T_{2}^{k},\ldots ,\mu_{m}^{k}\Delta T_{m}^{k}\right]}^{T}=-{\left(H+\lambda \;diag\left(H\right)\right)}^{-1}g,$$

where m is the number of the LRFs, k is the index of the iteration, $\mu_{j}^{k}\left(j=2,\ldots m\right)$ stands for the rotation increment represented by the exponential map $\left(e^{u_{j}}R_{j}\right)$, $\Delta T_{m}^{k}$ stands for the translation increment, λ is the Levenberg–Marquardt damping factor, H is the Hessian matrix (a symmetric matrix of dimension 6(m−1)), and g is the gradient (a column vector of dimension 6(m−1)) of the cost function. As for the specific calculation of H, g, and $\omega_{i}^{x}$, please refer to Reference [33]. After the solving using Levenberg–Marquardt, the rotation matrix is updated using the update exponential map [37] as

(12) $$R_{j}^{k+1}=e^{\mu_{j}^{k}}R_{j}^{k},\;T_{j}^{k+1}=\Delta T_{j}^{k}+T_{j}^{k},\;j\in \left[2,m\right]$$

from an initial guess for the relative poses of LRFs, which may be obtained from a rough measurement of the rig.

## 4. Experiments and Analysis

### 4.1. Simulation

Simulation experiments were based on the LRF scanning geometry model and the corridor geometry model. By setting the initial poses for LRFs and the rotational motion trajectory time series of the device as the input of the simulation, the calibration result could be gotten. The true LRF poses were set in the scanning geometry, so they could be taken as the true values in evaluating the calibration accuracy.

The scanning model of LRFs is based on HOKUYO UTM-30LX 2D LRF, whose characteristics are shown in Table 1. In order to make the simulated observation data close to the actual data, Gauss noise with *σ* = 0.03 m [38] was added to the simulated data.

Rotation operation is important for the calibration because it is directly related to whether the collected data has sufficient geometric constraints. Considering that some devices may be heavy rather than very convenient for rotating in actual use, Table 2 lists several sets of rotations that can be simulated and applied in almost all situations. The device pose in operation F (random pose) at each moment in the simulation system was random, but it could be used as a random rotation operation in reality. In addition, the rotation sequence of the Euler angles was “*z-x-y*” (yaw-pitch-roll), and the start pose of the device was the same as the world reference pose in Figure 2b.

**Figure 7:**
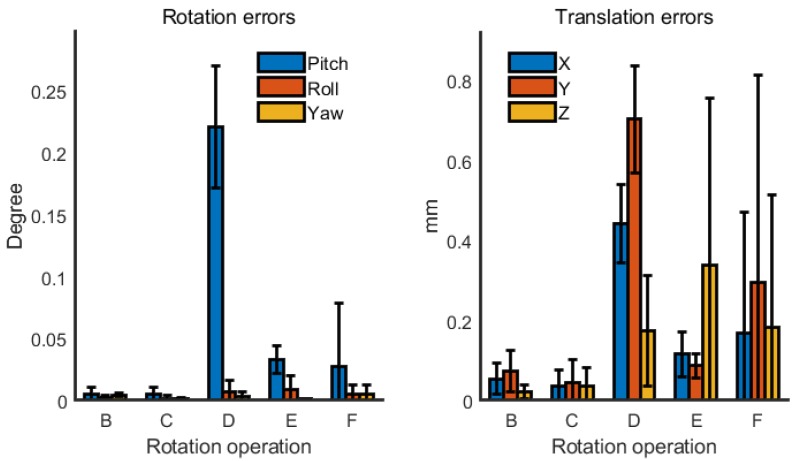
Error distribution of simulation based on different rotation operation.

The geometric model of the corridor was set to a simple rectangular tubular structure with an infinite length. First, the width and height of the corridor were set to 2 m. The simulated true poses of LRF_2_ and LRF_3_ were set to [−80°, 0°, −35°], [−150 mm, 150 mm, −200 mm], and [80°, 0°, −150°], [150 mm, 150 mm, −500 mm], respectively, whereas their simulated initial poses were set to [−90°, 0°, −30°], [−100 mm, 100 mm, −250 mm], and [90°, 0°, −145°], [100 mm, 100 mm, −550 mm], respectively. Each operation gathered 360 frames of data, which was taken as the input of the calibration process. After 10 repeated calibrations (the simulated data was regenerated at each repeat), the error distribution of the calibrated three Euler angles and three translation values of LRF_2_ based on operations B, C, D, E, and F were calculated and are shown in Figure 7, but not the results based on operation A.

Since operation A was only the rotation of the device in the yaw angle and without pitch angle or roll angle, the data of each frame of LRF_1_ could only scan the wall on the left and right sides, so only two parallel straight lines were obtained, which could not provide enough constraints. After the 10 repeated calibrations, the mean value of the calibrated Euler angles and three translation values of LRF_2_ were [−78.56°, 4.43°, −34.89°] and [−159.16 mm, 161.94 mm, −184.25 mm], which deviated from the set true roll angle by more than 4° and deviated from the set true *z* translation by more than 15 mm.

At the same time, the calibration results based on the operations B, C, D, E, and F were very close to the true value. Although the accuracy based on the five operations appeared to be lightly different from Figure 7, in fact the ease of the operation could be considered more. For example, operation C and operation D were too complicated, so they are not recommended in general. However, if the device is too large or inconvenient to rotate 360°, operation E can be considered. Of course, the actual operation is not limited to these, as long as it is capable of providing sufficient constraints.

**Figure 8:**
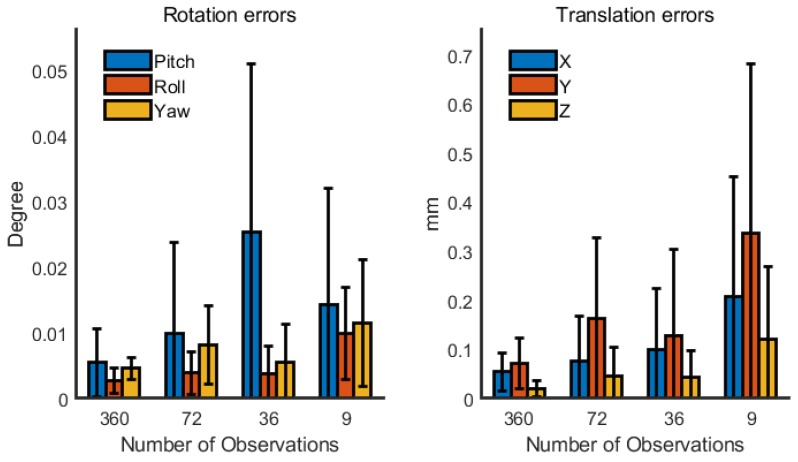
Error distribution of simulation with different numbers of observations based on operation B.

The error distribution of 10 repeated simulation experiments with different numbers of observations are shown in Figure 8. Based on operation B, frame sample intervals of 1, 5, 10, and 40 were set to get 360, 72, 36, and 9 observations, respectively, from the original generated 360 frames of simulated data. As can be seen from the figure, in general, the greater the number of observations, the higher the accuracy of the calibration results were.

**Figure 9:**
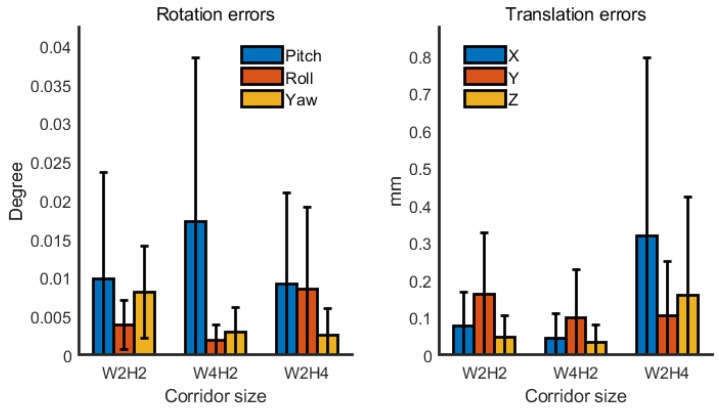
Error distribution of simulation with different corridor sizes based on operation B.

In order to verify that the method was applicable to corridors of different dimensions, the corridor dimensions with width to height ratios of 1:1, 2:1, and 1:2 were each set under the simulation experiment. The simulated corridor width and height corresponding to these ratios were [2 m, 2 m], [4 m, 2 m], and [2 m, 4 m]. Similarly, the error distribution after 10 repeated times based on operation B and a 5-frame sample interval is shown in Figure 9. Therefore, the different aspect ratios of the corridor had little effect on the calibration.

### 4.2. Real Data

**Figure 10:**
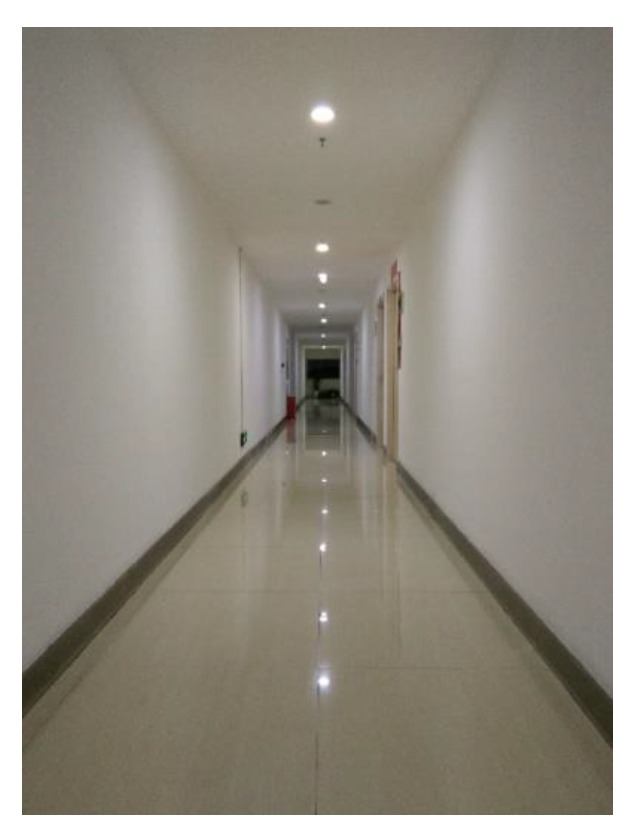
Experiment scene A.

The 3D indoor mapping device shown in Figure 1 was taken as the calibration device. The device was placed on a trolley to easily do the rotation movements. Figure 10 shows the experiment environment, which is easy to find in many buildings, such as an office building. The device employed three HOKUYO UTM-30LXes (the characteristics of the LRF are shown in Table 1), and the extrinsic calibration of LRF_1_ and LRF_2_, and LRF_1_ and LRF_3_, were done automatically after the gathering of data.

**Figure 11:**
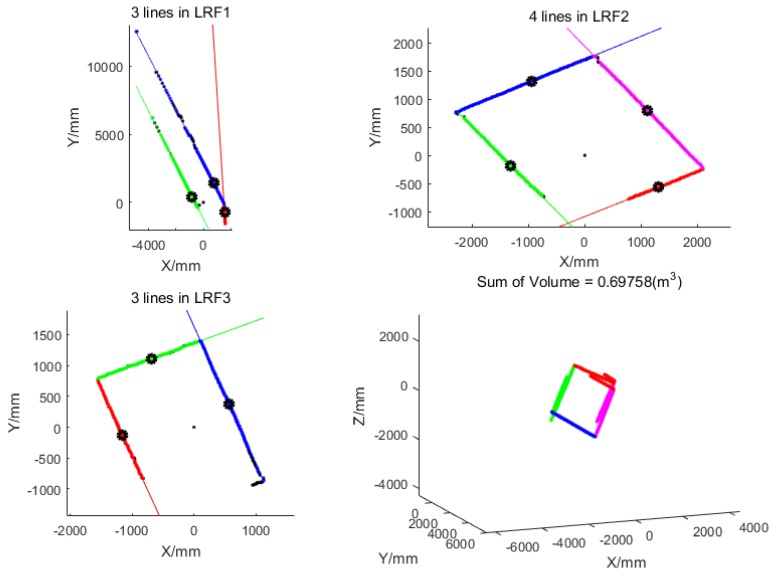
The line detection results and the selected correct corridor observations. Left-top, right-top, and left-bottom are the line detection results of LRF_1_, LRF_2_, and LRF_3_, respectively, and the sequence of the lines are plotted by red, green, blue, and magenta. Right-bottom is the corresponding correct corridor observation based on the initial poses (from the view of the corridor direction). The lines at the same surface are plotted by the same color, and the order of colors is still red, green, blue, and magenta.

Figure 11 shows an example of the line detection result and the correct corridor observation selection result. The color of the lines should be noted. The method in Section 3.2 was used to sort the lines detected in each LRF, and they are displayed in red, green, blue, and magenta in order. It is apparent from the right-down figure in Figure 11 that the line segments scanned by the three LRFs could not be perfectly overlapped into a rectangle based on the initial extrinsic parameters.

**Figure 12:**
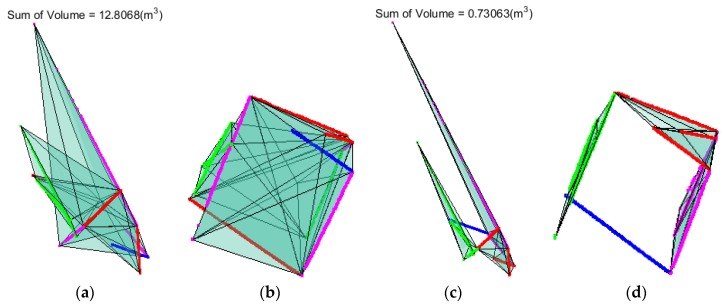
The comparison of the sum of the volume of tetrahedrons in an incorrect corridor observation and in a correct corridor observation. (**a**,**b**) The different views of the tetrahedrons based on an incorrect corridor observation; (**c**,**d**) the different views of the tetrahedrons based on a correct corridor observation. The lines on the same surface are plotted by the same color. It should be noted that there was no tetrahedron when there were fewer than two lines on a surface.

Figure 12 shows the contrast between an incorrect corridor observation and a correct corridor observation with the same group of data and the same initial poses of LRFs. When the observation was not the correct one, which means that the “coplanar lines” were not in fact on the same surfaces, the sum of the volumes of all the tetrahedrons was greatly larger than the correct one. Hence, with this assessment method, the inputted correct coplanar line pairs and neighboring surface pairs could be ensured to be correct as long as the initial poses were not far from the true poses.

**Figure 13:**
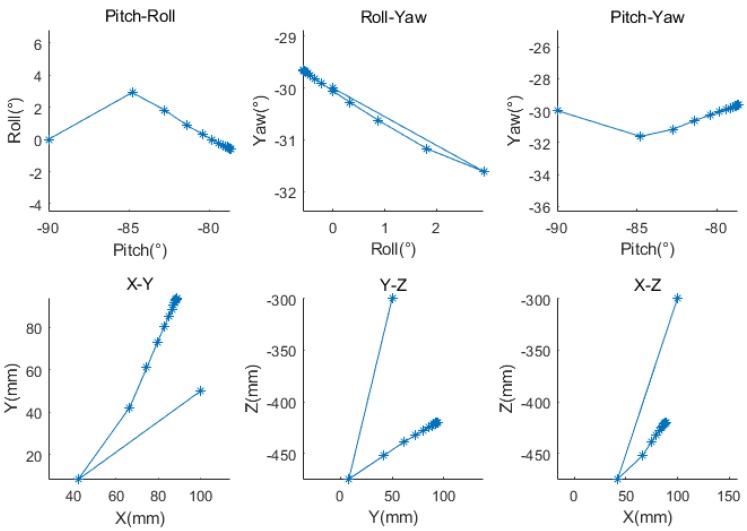
The convergence trajectory of the calibration parameters.

The initial poses of LRF_2_ and LRF_3_ were set to [−90°, 0°, −30°], [100 mm, 50 mm, −300 mm], and [90°, 0°, −140°], [−100 mm, 50 mm, −600 mm], respectively. Figure 13 shows the update trajectories of the three Euler angles and the three translations of LRF_2_ during calibration. The magnitude of the parameter update gradually decreased and gradually approached the target solution as the iteration progressed.

**Figure 14:**
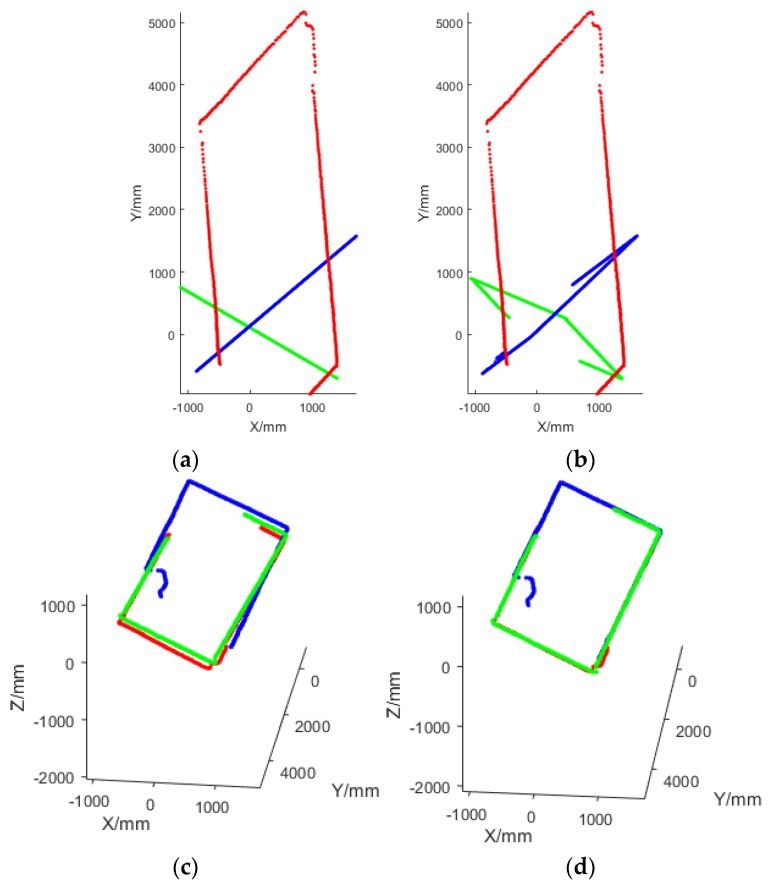
Comparison of the fused point cloud before and after calibration. (**a**,**c**) The different views of the fused point cloud before the calibration; (**b**,**d**) the different views after the calibration. The points from LRF_1_, LRF_2_, and LRF_3_ are plotted by red, green, and blue, respectively.

The purpose of the extrinsic calibration was to fusion all the point clouds from multiple LRFs into a global reference frame. Because the corridor was cuboid-shaped, if the calibration result was accurate, then projecting the fused point cloud onto a plane perpendicular to the corridor was a rectangle formed by a plurality of 3D line segments overlapping together. Figure 14 shows the comparison before and after calibration in this case. Therefore, after the calibration, the point clouds from three LRFs could be well merged into a cuboid-shaped corridor, so that the accuracy of the calibration result could be visually verified.

**Figure 15:**
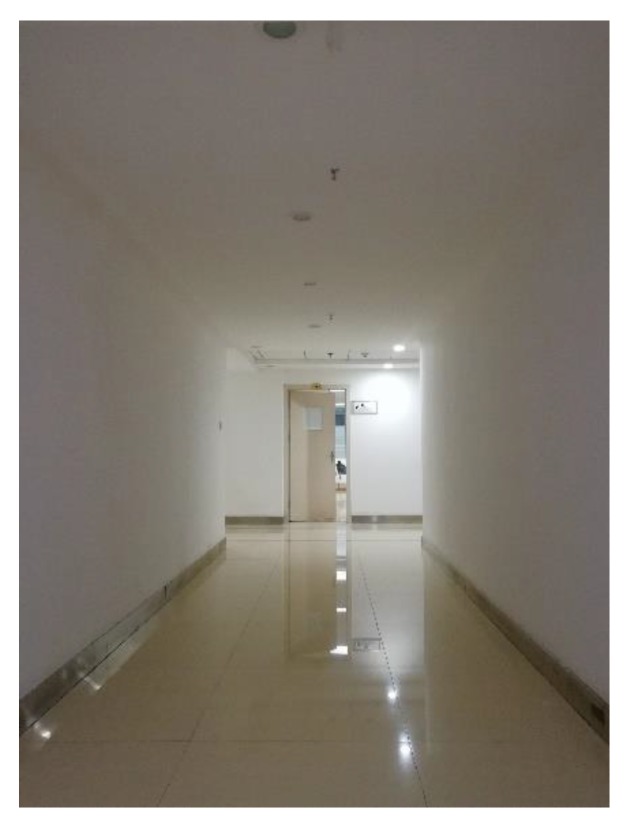
Experiment scene B.

To verify the repeatability of the method, more experimental results are shown in Table 3 and Table 4. Among them, Table 3 is the calibration result based on the device in Figure 1 and experiment scene A, and a total of three sets of data with each set of data was repeatedly calibrated 10 times to obtain the average value and standard deviation of the results. Considering that some devices are large like the device in Reference [39], they may be inconvenient to rotate in small scenes like scene A. Therefore, the configuration of the three LRFs was reconfigured and then recalibrated in scene B (Figure 15). The width and height of the corridor in scene A were 1.7 m and 2.4 m, respectively. Because the corridor was long enough, the three datasets in Table 3 were gathered based on operations B, C, and F. The length of the corridor in scene B was only 6.4 m, and its width and height were 2.2 m and 2.3 m, respectively. Thus, the three datasets in Table 4 were gathered based on operation E.

The initial poses of the LRFs in Table 3 were the same as above, and the initial poses of LRF_2_ and LRF_3_ in Table 4 were set to [90°, 0°, −155°], [−100 mm, 150 mm, −150 mm], and [90°, 0°, 155°], [100 mm, 150 mm, −1200 mm], respectively. As can be seen from Table 3 and Table 4, the deviation of the calibration result was kept substantially within 1° and 10 mm. In addition, it could be found that the deviation of the pitch angle was larger than the deviation of the roll angle and the yaw angle, and the deviation of the translation in the *x* direction was larger than the deviation of the translation in the *x* direction and the translation in the *z* direction. This phenomenon was normal for Table 3 because it was the same as the error distribution based on operation B, C, and F (simulation results in Figure 7). But for Table 4, it was because it was difficult to provide a relatively large pitch angle for the device in actual operation, so that the constraint in the *y* direction was weak, and the constraint in the pitch angle was also weak. Thus, the error distribution was more like the error distribution based on operation B.

**Figure 16:**
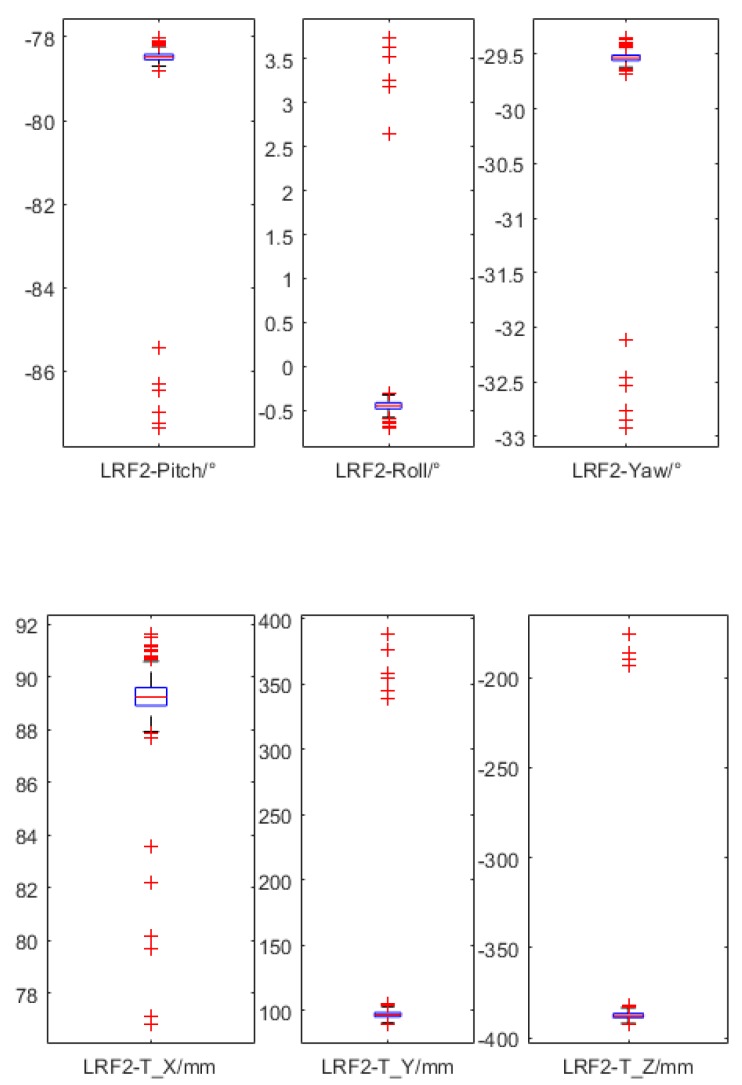
The calibration result distribution of LRF_2_ based on different initial poses.

To further prove the stability of the method, two batches of calibration experiments were implemented based on the first dataset in Table 3. First, almost all the conditions of the mismeasurement by hand for the initial poses of LRFs were taken into consideration. Assuming that each of the initial Euler angles had three possibilities with deviations of −10°, 0, and +10°, and each of the initial translations had three possibilities with deviations of −100 mm, 0 mm, 100 mm, then there were $3^{6}=729$ combinations of all considered deviation possibilities. Taking the calibration of LRF_2_ as an example, the referenced accurate pose could be gotten from Table 3, and the result distribution of the 729 times of calibration is shown in Figure 16. There were only 19 outliers among all the results, with most of them centered on a small range. In addition, from a practical point of view, the 10° and 100 mm error could be avoided by a manual measurement.

**Figure 17:**
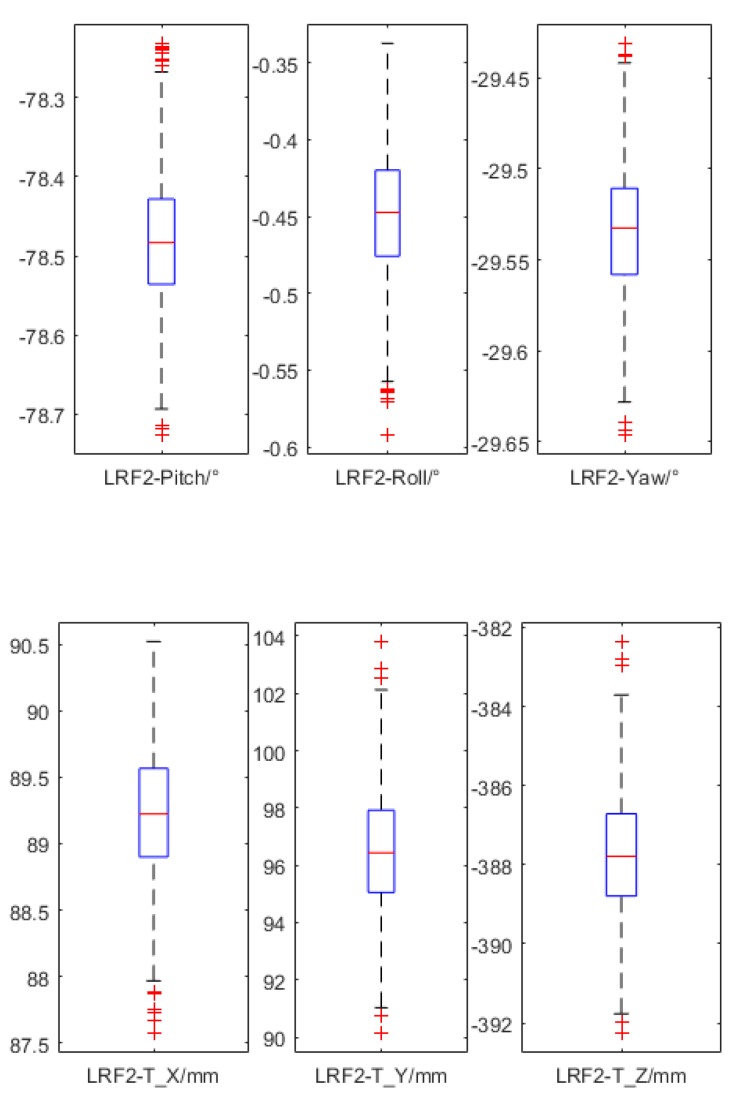
The calibration result distribution of LRF_2_ based on the same accurate initial pose.

Furthermore, another 729 times of calibration were implemented without deviations, which meant all the initial extrinsic parameters came from the centered results in Figure 16. As shown in Figure 17, the calibration results were all stably distributed around an accurate result. Hence, the calibration method was stable under multiple tests.

## 5. Discussion

The proposed extrinsic calibration method of 2D LRFs uses a common cuboid-shaped corridor as the experiment environment, which is very common in indoor buildings. The method in Reference [33] was not very suitable for indoor devices due to corners that can meet the requirement being scarce in most of cities, especially in indoor environments. Whether it is a corner or a corridor, it should be of sufficient length and have no material that reflects light or transmits light on its surfaces. Obviously, this method is more suitable under indoor conditions.

Although the method of solving nonlinear optimization problems and the geometric constraints used are the same as the method in Reference [33], the calibration scene used and the processes from the acquiring of the data to the finding of corridor observations are different. First, as for the calibration scenes, the authors in Reference [35] made two extrinsic calibration solutions, one of them based on scanning a flat plane and another one based on scanning perpendicular planes. The former one needed at least three LRFs, and the latter one needed two or three perpendicular planes. The method in this paper is based on scanning a cuboid-shaped corridor, which is with four planes. Second, the line detection in this method was modified based on traditional RANSAC as well as the coplanarity assessment method, and the corridor assessment method was proposed to make sure the inputted observations for the nonlinear solver are correct, resulting in a stable calibration result. These methods can also be extended to other application scenarios.

It should be noted that the device cannot be rotated too fast in actual operation. Since the 2D LRF ranges based on the rotation of a laser beam, each laser point on the scanning plane is not obtained at the same time as other laser points. Thus, if the device moves or rotates fast, the gathered points in each frame are not the same 3D plane (it is the same time in simulation). This is one of the sources of the calibration error, actually. If it moves or rotates too slowly, the amount of data collected is very large, and then frame sampling is required when the calibration is performed, or otherwise the processing time is long. Therefore, the speed of the rotating should not be fast, but it does not need to be too slow.

Future work may focus on reducing the method’s requirements for accuracy of the initial poses. In general, manual measurement errors are generally guaranteed to be within 10 cm because the three translation parameters are independent of each other. However, since the three Euler angles are not independent of each other, even the different Euler angle combinations can generate the same orientation matrix, so if the method of manually measuring the pose angles is inappropriate, it is likely to cause the initial angles to deviate more than 10°.

Moreover, devices equipped with multiple LRFs are often equipped with an IMU, just like the one shown in Figure 1, and the extrinsic calibration of the IMU is often necessary. After the extrinsic calibration of LRFs, the relative pose relationship between them is determined so that they can be considered as a rigid sensor, meaning that they can be used as a 3D laser scanner together. Thus, the calibration of the extrinsic parameters between the IMU and the LRFs is easier than the calibration of the extrinsic parameters of the IMU and a single LRF.

## 6. Conclusions

An extrinsic calibration method for multiple 2D LRFs by using an existing cuboid-shaped corridor as the reference is proposed. It does not need special artificial targets in the environment and does not need supervised data association. Its only requirement is rotating the LRFs to scan a common indoor cuboid-shaped corridor.

The coplanarity constraint and the orthogonality constraint from an indoor corridor are used for finding the accurate relative poses of LRFs. In order to provide the nonlinear solver correct input, a modified RANSAC algorithm, the line sorting method, the coplanarity assessment method, as well as the corridor assessment method are proposed to improve the stability of the finding of the correct corridor observation. Among them, the modified RANSAC algorithm and the coplanarity assessment method may also be useful for other applications.

The following work may focus on improving the robustness of the initial pose deviation. This may be done by adding other useful geometric constraints. At the same time, based on this work and based on the result of this calibration, the extrinsic calibration of the IMU will also be a future research plan.

## Figures and Tables

**Table 1:** Characteristics of the HOKUYO UTM-30LX 2D laser rangefinder.

Detection Range	σ	Angular Resolution	Measurement Resolution	Field of View	Scan Speed
0.1–60 m	0.03 m	0.25°	0.001 m	270°	25 ms

**Table 2:** List of rotation operations under simulation: “sin()” is the sine function, and “rand(*m*,*n*)” is a function to generate an *m*-by-*n* matrix with numbers within [0, 1].

Operation Name	*t* Sequence	Pitch Sequence (°)	Roll Sequence (°)	Yaw Sequence (°)
A	1, 2, …, 360	0 × *t*	0 × *t*	*t*
B	1, 2, …, 360	0 × *t* + 45	0 × *t*	*t*
C	1, 2, …, 360	sin(4 × *t*) × 45 + 45	0 × *t*	*t*
D	1, 2, …, 360	sin(4 × *t*) × 45 + 45	sin(4 × *t*) × 45 + 45	*t*
E	1, 2, …, 360	(360 − *t*) × 45/360	0 × *t*	sin(4 × *t*) × 90
F (random pose)	-	rand(360,1) × 360	rand(360,1) × 360	rand(360,1) × 360

**Table 3:** Calibration results and their deviations based on scene A.

Item	Rotation (°)	Rotation Dev. (°)	Translation (mm)	Translation Dev. (mm)
No. 1: LRF2	−79.28, −0.63, −29.84	0.19, 0.05, 0.05	93.23, 97.27, −405.35	0.55, 6.85, 3.29
No. 2: LRF2	−78.31, −1.11, −29.57	0.26, 0.14, 0.10	94.79, 91.65, −405.15	0.57, 4.51, 3.27
No. 3: LRF2	−78.82, −0.84, −29.72	0.43, 0.23, 0.14	93.42, 99.60, −406.79	0.98, 5.81, 3.73
No. 1: LRF3	85.16, 1.14, −135.72	0.15, 0.04, 0.03	−173.31, 59.24, −622.66	1.76, 3.04, 1.45
No. 2: LRF3	85.28, 1.24, −135.63	0.20, 0.05, 0.04	−171.33, 54.08, −624.87	2.33, 3.73, 1.64
No. 3: LRF3	85.46, 1.27, −135.55	0.14, 0.06, 0.05	−169.73, 52.64, −624.38	1.73, 2.44, 1.50

**Table 4:** Calibration results and their deviations based on scene B.

Item	Rotation (°)	Rotation Dev. (°)	Translation (mm)	Translation Dev. (mm)
No. 1: LRF2	90.31, 1.86, −157.87	0.31, 0.10, 0.05	−97.7, 208.93, −1355.90	1.30, 6.96, 3.54
No. 2: LRF2	90.24, 1.80, −157.72	0.27, 0.08, 0.02	−90.80, 211.66, −1350.00	1.56, 6.61, 4.67
No. 3: LRF2	89.80, 2.05, −158.55	0.39, 0.13, 0.01	−92.66, 212.95, −1350.50	1.68, 10.30, 5.00
No. 1: LRF3	85.51, 1.18, 162.56	0.18, 0.06, 0.05	103.97, 212.24, −987.95	0.48, 2.11, 2.09
No. 2: LRF3	86.16, 1.67, 162.99	0.16, 0.06, 0.04	109.41, 211.43, −975.19	0.42, 1.92, 2.83
No. 3: LRF3	86.38, 1.53, 162.44	0.51, 0.13, 0.05	104.68, 205.66, −976.37	1.30, 6.96, 3.54
